# Photoaging as a Social Marker and the D.I.F.A. Assessment: Addressing Social Stratification, the Filtered Ideal, and Identity Preservation in Brazil

**DOI:** 10.1111/jocd.70700

**Published:** 2026-01-29

**Authors:** Rafael Rodrigo Crisanto de Oliveira

**Affiliations:** ^1^ Centro de Ciências da Saúde Universidade Federal da Paraíba João Pessoa Paraíba Brazil

## 1 Introduction

In countries with intense solar irradiation such as Brazil, the skin operates simultaneously as a biological organ, a phenotypic marker of aging, and a visible marker of social belonging [1]. Marks produced by chronic photoexposure acquire meanings that extend beyond cutaneous physiology. They become embedded in social, economic, and symbolic dynamics that influence behavior, aesthetic expectations, and subjective experiences of aging. Amid ongoing ethical debates on whether aging should be viewed as pathological or socially constructed, it is relevant to examine how photoaged skin in Brazil may function as a visible stigma linked to social class and structural inequality [2, 3]. This phenomenon is deeply rooted in the structural hierarchy of labor and racialized class stratification, where cutaneous health outcomes are inextricably linked to systemic inequalities that determine environmental exposure and access to protective resources [4].

## 2 Clinical Phenotypes and the Paradox of Refinement

Aesthetic demands related to photoaging in clinical practice tend to cluster into two socioclinically distinct phenotypes. However, these are not rigid categories; the high degree of ethnic heterogeneity and fluid social mobility in Brazil often result in intermediate or hybrid clinical presentations, requiring a nuanced diagnostic approach [4, 5].

- Phenotype I (Structural Actinic Damage): This encompasses individuals exposed to intense and prolonged occupational sunlight, often with limited historical access to photoprotection. Clinical presentation includes pronounced dermal elastosis, multiple actinic keratoses, and irregular hyperpigmentation [1, 2]. These findings represent not only cumulative biological damage but also socioeconomic vulnerability. Therapeutic demand frequently transcends aesthetics, reflecting attempts to mitigate the social stigma associated with outdoor labor, which directly influences perceptions of employability and social integration [4, 6].
- Phenotype II (Digital Aesthetic Refinement): This includes individuals with consistent access to indoor environments and high aesthetic expectations. The predominant clinical presentation consists of subtle findings—fine lines and textural irregularities—driven by the pursuit of “glassy,” uniform skin influenced by digitally filtered standards [7, 8]. In this group, motivation is often fueled by “Snapchat Dysmorphia” [7].

A significant clinical paradox emerges: while these patients pursue idealized digital textures, they frequently present with Fear of Overfilling (FOF) [9], a clinically meaningful anticipatory anxiety frequently observed in aesthetic practice centered on iatrogenic identity loss that acts as a psychological barrier to treatment. The coexistence of these phenotypes reveals an ethical dilemma for cosmetic dermatology: while one population seeks to reduce stigmata linked to structural inequality, the other navigates a tension between unrealistic digital ideals and the fear of clinical distortion [9].

## 3 Digital Influence, Perception Drift and Public Health

Sun exposure in Brazil is marked by ambivalence. While culturally associated with leisure, it often reflects occupation and unequal access to care [1, 2]. Conversely, smooth skin signals aesthetic capital. Treating photoaging may partially reduce perceived inequalities, while lack of access tends to reinforce them [2, 8, 10]. At the same time, the hypervisibility of distorted results in digital environments has reshaped public perception. The widespread use of filters modifies subjective perceptions of normality [3], while “Zoom dysmorphia” has intensified self‐scrutiny [4, 5]. Among clinicians, repeated exposure to filtered demands may induce “perception drift,” where normal variations are reinterpreted as defects [8].

Furthermore, the normalization of Overfilled Face Syndrome (OFS) in social media reinforces FOF, challenging the therapeutic alliance as patients overestimate the risk of unnatural outcomes [9, 11]. Beyond the aesthetic and psychological dimensions, it is imperative to acknowledge that photoaging, regardless of social class, is the primary clinical precursor to skin cancer. Both phenotypes are subject to the cumulative and deleterious effects of ultraviolet radiation, which remains a critical public health concern in Brazil due to the high incidence of basal cell carcinoma, squamous cell carcinoma, and melanoma [6].

## 4 The D.I.F.A. Framework: A Proposed Clinical Tool

To address these complexities, the implementation of the D.I.F.A. (Digital Influence & Filter Assessment) is proposed as a non‐pathologizing communication framework. This tool is intended not as a diagnostic scale, but as a pragmatic framework derived from clinical observation to guide ethical decision‐making. By addressing perceptual distortion before intervention planning, the D.I.F.A. contributes to the demedicalization of normality and the reduction of overtreatment risk. Distinct from Body Dysmorphic Disorder (ICD‐11: 6B21) [12], this assessment targets subclinical perception drift through four structured steps:

1. Source Analysis: Identification of the patient's primary aesthetic references, distinguishing between achievable biological restoration and algorithm‐driven distortions [3, 7, 13].
2. Real‐Reflection Comparison: Contrast of digital filters with the patient's biological reflection under standardized clinical lighting to recalibrate the internal reference point for naturalness [4, 5].
3. Clinical Expectation Alignment: Validation of physiological skin texture and establishment of realistic therapeutic endpoints. This step includes a comprehensive clinical screening for actinic malignancies, reinforcing that the primary goal of dermatologic intervention is the maintenance of cutaneous health and cancer prevention [6, 8].
4. Fear of Overfilling (FOF) Screening: Explicit screening for Fear of Overfilling (FOF) [9] to identify identity‐related anxieties. This allows for the strategic redirection of the treatment plan toward structural restoration and bioregenerative modalities (e.g., PDRN and lasers), ensuring the preservation of essential facial character while improving skin rheology without unintended volume [9].

## 5 Conclusion

Photoaging in Brazil is largely a socially stratified dermatologic phenomenon. Clinical management thus has a dual mission: preventing malignancy while reducing the symbolic burden of occupational exposure. Within this scenario, the dermatologist functions as a steward of facial identity. By integrating the D.I.F.A. framework and addressing FOF, the clinician can mitigate defensive attitudes and support patient confidence in safe rejuvenation, promoting an aesthetic grounded in health, diversity, and authenticity. Naturally, class‐related stratification of photoaging does not imply determinism, and individual variance remains substantial.

Identity preservation refers not to resisting rejuvenation, but to preserving facial recognizability, proportional coherence, and cultural self‐perception within socially realistic expectations. By framing photoaging as both a biological condition and a socially stratified phenomenon, this commentary invites clinicians to move beyond purely technical intervention and engage with the psychosocial and oncological realities that shape aesthetic demand.

## Ethics Statement

The author confirms that the ethical policies of the journal, as noted on the journal's author guidelines page, have been adhered to. No ethical approval was required as this is a review article with no original research data.

## Conflicts of Interest

The author declares no conflicts of interest.

## Data Availability

The data that support the findings of this study are available on request from the corresponding author. The data are not publicly available due to privacy or ethical restrictions.
